# An Open Label, Randomised Trial of Artesunate+Amodiaquine, Artesunate+Chlorproguanil-Dapsone and Artemether-Lumefantrine for the Treatment of Uncomplicated Malaria

**DOI:** 10.1371/journal.pone.0002530

**Published:** 2008-06-25

**Authors:** Seth Owusu-Agyei, Kwaku Poku Asante, Ruth Owusu, Martin Adjuik, Stephen Amenga-Etego, David Kwame Dosoo, John Gyapong, Brian Greenwood, Daniel Chandramohan

**Affiliations:** 1 Kintmapo Health Research Centre, Ministry of Health, Kintampo, Ghana; 2 Infectious and Tropical Diseases Department, London School of Hygiene & Tropical Medicine, London, United Kingdom; 3 Navrongo Health Research Centre, Ministry of Health, Navrongo, Ghana; 4 Heatlh Research Unit, Ministry of Health, Accra, Ghana; Faculty of Tropical Medicine, Mahidol University, Thailand

## Abstract

**Background:**

Artesunate+amodiaquine (AS+AQ) and artemether-lumefantrine (AL) are now the most frequently recommended first line treatments for uncomplicated malaria in Africa. Artesunate+chlorproguanil-dapsone (AS+CD) was a potential alternative for treatment of uncomplicated malaria. A comparison of the efficacy and safety of these three drug combinations was necessary to make evidence based drug treatment policies.

**Methods:**

Five hundred and thirty-four, glucose-6-phosphate dehydrogenase (G6PD) normal children were randomised in blocks of 15 to the AS+AQ, AL or AS+CD groups. Administration of study drugs was supervised by project staff and the children were followed up at r home on days 1,2,3,7,14 and 28 post treatment. Parasitological and clinical failures and adverse events were compared between the study groups.

**Main Findings:**

In a per-protocol analysis, the parasitological and clinical failure rate at day 28 post treatment (PCF28) was lower in the AS+AQ group compared to the AL or AS+CD groups (corrected for re-infections: 6.6% vs 13.8% and 13.8% respectively, p = 0.08; uncorrected: 14.6% vs 27.6% and 28.1% respectively, p = 0.005). In the intention to treat analysis, the rate of early treatment failure was high in all three groups (AS+AQ 13.3%; AL 15.2%; and AS+CD 9.3%, p = 0.2) primarily due to vomiting. However, the PCF28 corrected for re-infection was lower, though not significantly, in the AS+AQ group compared to the AL or the AS+CD groups (AS+AQ 18.3%; AL 24.2%; AS+CD 20.8%, p = 0.4) The incidence of adverse events was comparable between the groups.

**Conclusions:**

AS+AQ is an appropriate first line treatment for uncomplicated malaria in Ghana and possibly in the neighbouring countries in West Africa. The effectiveness of AL in routine programme conditions needs to be studied further in West Africa.

**Trial Registration:**

ClinicalTrials.gov NCT00119145

## Introduction

There is now widespread acceptance that combination therapy (≥2 antimalarial drugs with different modes of action) is a better option than monotherapy for the treatment of uncomplicated malaria and artemisinin based combination therapy (ACT) is advocated as a way forward.[1] The WHO recommends artesunate+amodiaquine (AS+AQ), artemether-lumefantrine (AL) or artesunate+sulphadoxine-pyremethamine (AS+SP) as the first line treatment for uncomplicated malaria in Africa.[2] By November 2006, most malaria endemic countries in Africa (33/42) had adopted ACT as the first line treatment for uncomplicated malaria.[3] In 2002, Ghana adopted AS+AQ as first line treatment based on the evidence of its efficacy and safety observed elsewhere in Africa and on account of its potential for production by local manufacturers.[4] Randomised controlled efficacy trials in Zanzibar,[5] Uganda[6] and Angola[7] had shown that polymerase chain reaction ( PCR) corrected adequate clinical response rates at day 28 post treatment (ACPR28) were very high for both AS+AQ (94–100%) and AL (97–100%). In 2003, a four arm comparative efficacy trial in Ghana showed that the ACPR28 was 100% (n = 51) for AL and 97.5% (n = 54) for AS+AQ.[8] However, a randomised trial of the effectiveness of AS+AQ and AL in Tanzania showed that the parasitological failure at day 28 was 2.6 fold (95% CI 1.9–3.4) higher in the AS+AQ group (40%) compared to the AL group (21%).[9] Artesunate+chlorproguanil-dapsone (AS+CD) is another potential alternative for AS+AQ because chlorproguanil-dapsone has been shown to be efficacious in areas with high SP and chloroquine resistance.[10] Clinical trials of AS+CD have been carried out in several countries in Africa (www.ispub.com/ostia/index.phpxmlFilePathjournals/ijid/vol4n2/cda.xml) but the development of AS+CD was abandoned recently due to safety concerns in glucose-6-phosphate dehydrogenase (G6PD) deficient subjects. There has been no comparative studies of the efficacy and safety of AS+CD versus AS+AQ or AL. Even though most countries in Africa have adopted ACT as first line treatment, antimalarial monotherapy is still common and this can undermine the efficacy of ACT. Furthermore high levels of resistance to AQ already exists in many parts of Africa.[9] Thus, it is important to evaluate the efficacy of ACTs particularly that of AS+AQ periodically. In this paper, we report the results of a randomised trial that compared the efficacy and safety of AS+AQ, AL and AS+CD in children in Ghana.

## Methods

The protocol for this trial and supporting CONSORT checklist are available as supporting information; see checklist S1 and Protocol S1.

### Participants

**Figure 1:**
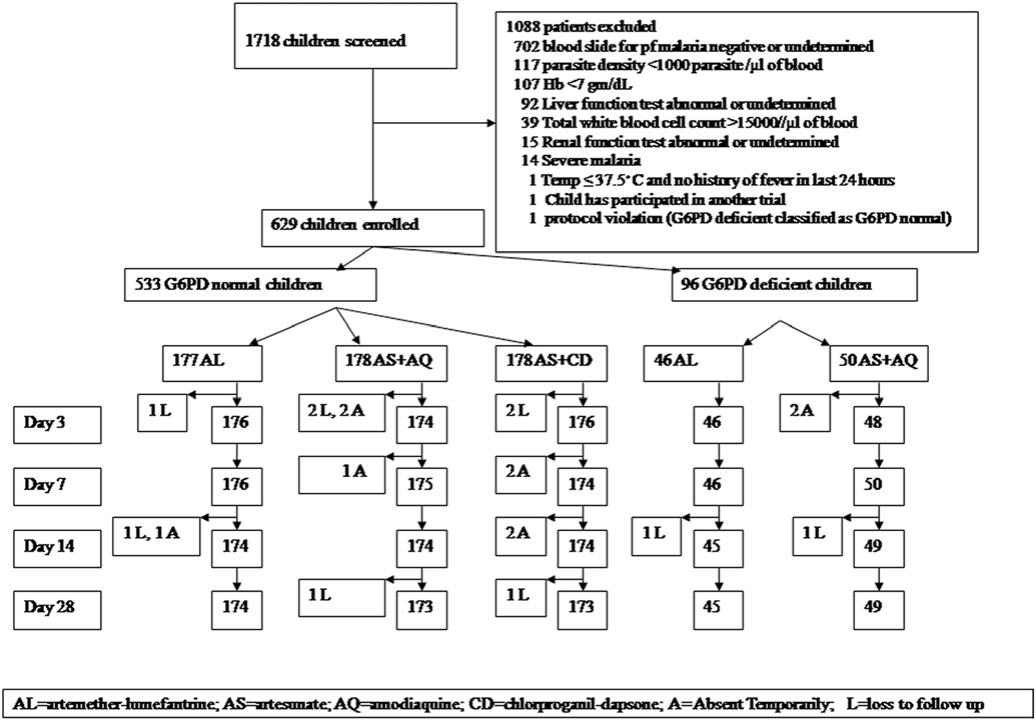
Enrolment and Follow up profile.

The study was conducted in Kintampo district hospital, Brong-Ahafo region, Ghana. Malaria transmission in this forest region of Ghana is perennial but peaks in July–August and the entomological inoculation rate is high, about 270 infective bites per person per year. Although the study area has high transmission, malaria is common in older children and therefore the study included children up to 10 years of age. From June 2005 to May 2006, 1718 children aged 6 months to 10 years who attended the study hospital's outpatient department with a history of fever or an axillary temperature ≥37.5°C were examined for malaria parasitaemia and screened for their eligibility for enrolment in this study after written, informed consent had been obtained from their caretakers (Figure 1). Children who had a body weight ≤5 kg, Hb <7 g/dL, malaria parasitaemia <2000 or >200000 parasites/μL, danger signs (unable to drink, history of repeated vomiting, convulsions or other signs of severe malaria, or any other concomitant febrile disease were excluded from the study but offered standard care according to Ministry of Health guidelines. Children were screened for glucose-6-phosphate dehydrogenase (G6PD) deficiency before randomisation (the G6PD test took about 1hour). A total of 630 children were eligible; 534 were G6PD normal and 96 were G6PD deficient. The G6PD normal children were randomly assigned to the AL, AS+AQ, or AS+CD groups. The G6PD deficient children were randomised to the AS+AQ or AL group only because the Ghana Ministry of Health Ethical Committee expressed concern about the possibility of haemolysis associated with chlorproguanil-dapsone treatment in G6PD deficient children.

### Procedures

Administration of the first dose of study drugs was supervised by a study nurse and the administration of subsequent doses was supervised by study field workers. All children were given iron along with antimalarials according to national treatment guidelines. Caretakers of children in the AL group were told about the need to give a fatty meal along with the drug. Children were actively followed up at their homes by 10 field workers on days 1, 2, 3, 7, 14 and 28 post treatment to solicit adverse events. Finger prick blood samples for parasitological observations were obtained on days 3, 7, 14, 28, for haematological measurements on days 1, 2, 3, 7, 28 and for biochemical assessments on days 2, 7, 28 (0.7ml of capillary blood) after the start of treatment. Blood samples were collected from children who attended the study clinic for suspected malaria on any other day outside the scheduled days of follow up. There were 77 unscheduled visits and those who had a positive blood slide on these unscheduled visits (n = 15) were treated with quinine and classified as treatment failures.

### Randomisation

Randomisation was done using Microsoft Excel 2003^©^ randomisation generator. G6PD normal children were randomised in blocks of 15 to the AL, AS-AQ, or AS+CD group. G6PD deficient children were randomised in blocks of 10 to AL or AS-AQ group. Co-blistered packs of artesunate and amodiaquine (Arsucam,® Sanofi-Aventis), artemether-lumefantrine (Coartem,^©^ Novartis) and chlorproguanil-dapsone (Lapdap,™ GlaxoSmithKline) were obtained from the respective local suppliers.

### Statistical Methods

Data were double-entered, and validated using Visual Foxpro^©^ 6.0.and the analysis was done using Stata™ 9.1. Per protocol analysis included patients who were properly randomised, had received the study drugs according to the protocol, and for whom data were available on the primary end point (ACPR28). Intention to treat (ITT) analysis involved all randomised patients irrespective of how many doses of the study drugs had been given but excluded all major day 0 protocol violations. All statistical tests were two-sided and an α-level <0.05 was considered a statistically significant result. For comparisons of continuous variables between groups, the t-test was used and for comparisons between more than two groups, one-way analysis of variance was used after assuring normality and homogeneity of variances assumptions were satisfied. For comparison of categorical variables, the chi-square test was used with the exact extension invoked when there were small numbers in the cells. A survival analysis using the life table method was done on the cumulative proportion of children having treatment failure at different time points during the post treatment period in each of the arms of the study. The end points were any of the following: failure to take the drug on any of the first three days, parasitaemia on day 2 greater than that on day 0, presence of parasitaemia on day 7, diagnosis of severe malaria at any point after day 0, recurrent parasitaemia after day 7 up to day 28. The likelihood-ratio test statistic of homogeneity and the logrank tests of homogeneity of arm were also carried out.

### Sample size

The primary endpoint was PCR corrected ACPR by day 28. A sample size of 510 children was chosen on the basis of the following assumptions: (1) the efficacy of AL would be 95%; (2) the study would have 80% power at 95% significance level to detect a 10% difference in efficacy as measured by the parasitological failure at day 28 post-treatment in the AS+AQ, and AS+CD groups compared with the AL group; (3) loss to follow up by day 28 post treatment would be <5%.

### Laboratory procedures

At least 200 oil immersion fields of thick blood films stained with Giemsa stain were examined for malaria parasites before a blood film was considered negative. The parasite density per micro-litre was estimated by multiplying the number of parasites per 200 leukocytes by a factor of 40, assuming a white blood cell count of 8,000/μl. Each blood slide was read by two microscopists. If the difference in the parasite density estimated by the two microscopists was <50%, the mean of the two readings was defined as the true parasite density. If the disagreement between the two readings was ≥50%, a third microscopist examined the slide and the mean of the two closest readings was deemed to be the true parasite density. To distinguish between recrudescent and new infections, parasites identified on Days 14 and 28 were compared to those identified at enrolment using PCR amplification of *msp2 P. falciparum* genes. Full blood count and biochemical tests were done by calibrated automated ABX MICROS 60 (Horiba ABX, France) and Selectra E Clinical Chemistry Analyzer (Vital Scientific N.V, Netherlands) respectively. Quantitative G6PD phenotype analysis was conducted using Randox method with Humalyser Junior (Human, Germany).

Plasma ALT, AST, Total Bilirubin, Creatinine and Urea were measured on the Selectra E Clinical Chemistry analyzer (Vital Scientific, The Netherlands) using reagents from Elitech Diagnostics (Sees, France). ALT and AST were measured using the kinetic, UV, IFCC method without pyridoxal phosphate, total bilirubin with the modified Evelyn-Malloy method, creatinine with the kinetic Jaffe method and urea with the kinetic urease method. Glucose-6-phosphate dehydrogenase (G6PD) activity was measured using a quantitative UV G6PD kit from Randox (Antrium, UK). The enzyme activity was determined by the measurement of the rate of absorbance change at 340 nm due to the reduction of NADP^+^.

### Ethics

The study protocol was approved by the ethics committees of the Kintmapo Health Research Centre, Ghana Ministry of Health and the London School of Hygiene & Tropical Medicine. The study is registered at the United States National Institute of Health clinical trials register; the registration number is NCT00119145 and the URL:https://clinicaltrials.gov/ct2/show/NCT00119145/

## Results

### G6PD normal children

There were no statistically significant differences in the demographic or baseline haematological characteristics between the three treatment groups at enrolment (Table 1).

**Table 1:** Demographic and haematological characteristics at enrolment in G6PD normal children.

Characteristics	AS+AQ [N = 178]	AL [N = 177]	AS+CD [N = 178]	P
	n (%)	n (%)	n (%)	
**Age (months)**				
6–11	26 (14.6)	25 (14.0)	22 (12.4)	1.0
12–59	119 (66.9)	120 (67.4)	122 (68.5)	
60+	32 (18.0)	33 (18.5)	34 (19.1)	
Mean (SD)	37.6 (24.3)	36.8 (26.2)	39.4 (25.0)	0.6
**Gender**				
Male	83 (46.9)	94 (52.8)	92 (51.7)	0.5
Female	94 (52.8)	84 (47.2)	86 (48.3)	
**Weight (Kg)**				
<9.9	50 (28.1)	54 (30.3)	46 (25.8)	0.8
10.0–19.9	117 (65.7)	112 (62.9)	118 (66.3)	
20.0+	10 (5.6)	12 (6.7)	14 (7.9)	
Mean (SD)	12.6 (4.3)	12.4 (4.5)	12.6 (4.3)	0.9
**Temperature (°C)**				
≥37.5	106 (59.6)	107 (60.1)	97 (54.5)	0.5
Mean (SD)	37.9 (1.1)	39.9 (1.1)	37.7 (1.1)	0.3
**Parasite Density/μL**				
<2500	39 (21.9)	31 (17.4)	32 (18.0)	0.2
2500-<10000	42 (23.6)	28 (15.7)	36 (20.2)	
≥10000	96 (53.9)	119 (66.9)	110 (61.8)	
Geometric mean (range)	12507 (1000, 198520)	16521 (1000, 197360)	13820 (1000,199280)	
Gametocytaemia	10 (5.6)	11 (6.2)	16 (9.0)	0.4
**Haemoglobin (g/dL)**				
6-<11	163 (91.6)	167 (93.8)	165 (92.7)	0.8
11+	14 (7.9)	11 (6.2)	13 (7.3)	
Mean (SD)	9.1 (1.4)	8.9 (1.3)	8.9 (1.4)	0.3

AS = artesunate; AQ = amodiaquine; AL = artemether-lumefantrine; CD = chlorproguanil-dapsone

**Figure 2:**
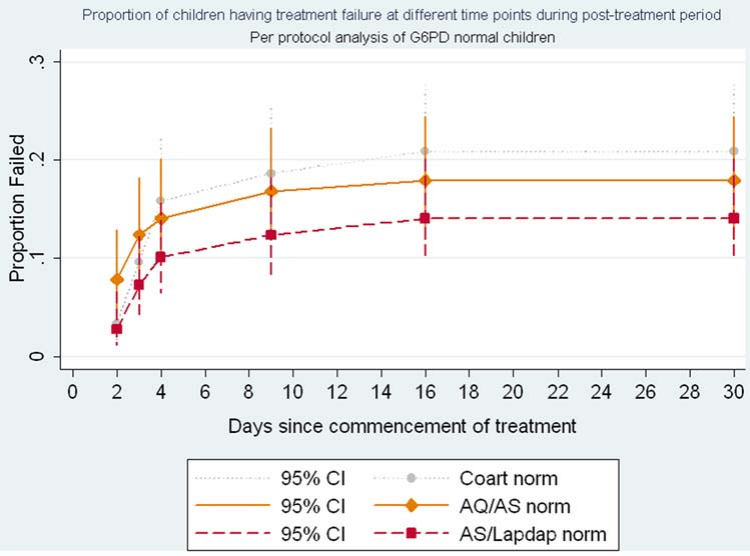
Proportion of children having treatment failures at different time points during the post treatment period Per protocol analysis of G6PD normal children.

In the per-protocol (PP) analyses there were no statistically significant differences in the rates of early treatment, late treatment, parasitological or clinical failures at day 14 between the three groups (Table 2). Survival analysis showed that there was no statistically significant differences in treatment failure at different time points during the 28 day post treatment period between the three groups (Figure 2). However, the parasitological and clinical failure rate at day 28 (PCF28) uncorrected for re-infections was significantly lower in the AS+AQ group (14.6%) compared to the AL (27.6%) or AS+CD (28.1%) groups (Table 2). The PCF28 corrected for re-infection was also lower in the AS+AQ group (6.6%) than in the AL (13.8%) or AS+CD (13.8%) groups but this difference was not statistically significant. PCF28 uncorrected for re-infection was nearly 50% less in the AS+AQ group compared to AL and AS+CD groups (Table 3). PCF28 corrected for new infections was significantly lower in the AS+AQ group compared to AS+CD group (RR 0.43; 95% CI 0.20, 0.89). There was no significant difference in the PCF28 between AL and AS+CD groups (Table 3).

**Table 2:** Treatment outcomes (per protocol analysis).

Outcomes	AS+AQ [N = 151]	AL [N = 152]	AS+CD [N = 160]	P*
	n (%)	n (%)	n (%)	
Early treatment failure	1 (0.7)	5 (3.3)	3 (1.9)	0.257
Late treatment failure	6 (4.0)	7 (4.6)	7 (4.4)	0.960
Parasitological or clinical failure by day 14	8 (5.3)	13 (8.6)	13 (8.1)	0.482
Parasitological or clinical failure by day 28 (PCR uncorrected)	22 (14.6)	42 (27.6)	45 (28.1)	0.005
Parasitological or clinical failure by day 28 (PCR corrected)	10 (6.6)	21 (13.8)	22 (13.8)	0.083
Missing PCR by day 28 (excluded)	3 (2.0)	9 (5.9)	13 (8.1)	-
Gametocytaemia by day 7	3 (2.0)	0 (0)	6 (3.8)	0.043

AS = artesunate; AQ = amodiaquine; AL = artemether-lumefantrine; CD = chlorproguanil-dapsone

* *exact P values*

**Table 3:** Comparison of treatment outcomes between groups (per protocol analysis).

Outcomes	AS+AQ vs AL	P*	AS+AQ vs AS+CD	P*	AS+CD vs AL	P*
	RR (95% CI)		RR (95% CI)		RR (95% CI)	
Parasitological or clinical failure by day 14	0.69 (0.28,1.63)	0.4	0.67 (0.27,1.55)	0.4	1.08 (0.49,2.22)	0.8
Parasitological of clinical failure by day 28 (PCR uncorrected)	0.53 (0.32,0.85)	0.007	0.51 (0.30,0.81)	0.004	1.01 (0.68,1.41)	1.0
Parasitological of clinical failure by day 28 (PCR corrected)	0.48 (0.23,0.98)	0.044	0.43 (0.20,0.89)	0.023	1.03 (0.57,1.47)	0.9

AS = artesunate; AQ = amodiaquine; AL = artemether-lumefantrine; CD = chlorproguanil-dapsone

* *since there are three comparisons Bonfferroni correction is applied; the P value has to <0.017 to be statistically significant*

In the intention to treat analyses, the rate of early treatment failure was high in all three groups (13.3% in AS+AQ, 15.2% in AL, and 9.3% in AS+CD groups respectively) but this was primarily due to vomiting twice or more within half an hour of administration of the study drugs. Among the early treatment failures, 22/23 in AS+AQ, 22/27 in AL, and 13/16 in AS+CD groups were due to repeated vomiting of study drugs. In the intention to treat analyses the relative risk (RR) of parasitological and clinical failure by day 28 corrected for reinfection was lower, though not statistically significantly, in the AS+AQ group compared to the AL group (RR 0.75; 95% CI 0.48, 1.14) or to the AS+CD group (RR 0.81; 95% CI 0.5, 1.27). The relative risk of parasitological and clinical failure corrected for reinfection was lower in the AS+AQ group compared to the AL (RR 0.68, 95% CI 0.47, 0.94) or AS+CD group (RR 0.72, 95% CI 0.49, 1.01).

There was a drop in mean Hb concentration on day 2 post treatment compared to day 0 in each of the three groups (Table 4). The mean drop in Hb on day 2 ranged from 0.8 g/dl in the AS+CD group to 0.4 g/dl in the AL group. By day 7 post treatment, the mean Hb concentration had recovered to the day 0 levels in the AS+AQ and AL groups and by day 28 the mean Hb was higher than the day 0 in all three groups. The rate of recovery of Hb was slightly slower in the AS+CD group compared to the other two groups.

**Table 4:** Haemoglobin concentration during the post treatment period in the study groups.

Post treatment day	AS+AQ (n = 151)	AL (n = 152)	AS+CD (n = 160)	P*
	Mean Hb g/dl (SD)	Mean Hb g/dl (SD)	Mean Hb g/dl (SD)	
0	9.0 (1.3)	9.2 (1.4)	8.9 (1.3)	0.2
1	8.5 (1.6)	8.7 (1.6)	8.3 (1.4)	0.08
2	8.3 (1.7)	8.8 (1.7)	8.1 (1.6)	0.002
3	8.8 (1.8)	8.9 (1.6)	8.4 (1.5)	0.045
7	9.0 (1.7)	9.3 (1.6)	8.6 (1.5)	0.001
28	9.9 (1.7)	10.0 (1.6)	9.7 (1.6)	0.3

AS = artesunate; AQ = amodiaquine; AL = artemether-lumefantrine; CD = chlorproguanil-dapsone

SD = standard deviation

* *since there are five time points compared against the baseline (day 0), Bonfferroni correction is applied; the P value has to <0.008 to be statistically significant*

The reported incidences of solicited adverse events during the 7 days post treatment are shown in Table 5. The incidence of solicited adverse events was comparable between the three groups. A history of body pain was more frequent in the AS+AQ group than the other two groups (Table 5).

**Table 5:** Distribution of solicited adverse events during the 7-day post treatment period.

Reported symptoms	AS+AQ (n = 178)	AL (n = 177)	AS+CD (n = 178)	P*
(Adverse events)	n (%)	n (%)	n (%)	
Unable to suck/drink	8 (4.5)	5 (2.8)	2 (1.1)	0.11
Fever	0 (0)	0 (0)	0 (0)	-
Runny nose	29 (16.3)	30 (17.0)	14 (7.9)	0.03
Cough	33 (18.5)	23 (13.0)	21(11.8)	0.30
Difficulty in breathing	3 (1.7)	4 (2.3)	2 (1.1)	0.48
Diarrhoea	13 (7.3)	19(10.7)	11(6.2)	0.44
Vomiting	8(4.5)	6(3.4)	12 (6.7)	0.58
Itching/pruritus	13 (7.3)	10 (5.7)	10 (5.6)	0.93
Loss of appetite	61 (34.3)	44 (24.9)	50 (28.1)	0.29
Nausea	2 (1.1)	0 (0.0)	5 (2.8)	0.17
Abdominal pain	31 (17.4)	19 (10.7)	28 (15.7)	0.34
Body pain	25 (14.0)	10 (5.7)	9 (5.1)	0.01
Difficulty in sleeping	2 (12.4)	23 (13.0)	16 (9.0)	0.75
Joint pain	4 (2.3)	1 (0.6)	0 (0.0)	0.28
Palpitation	3 (1.7)	4 (2.6)	5 (2.8)	0.89
Rash	2 (1.1)	3 (1.7)	2 (1.1)	0.97
Ulcers in mouth/tongue	15 (8.4)	12 (6.8)	10 (5.6)	0.82
Yellow eyes	4 (2.3)	3 (1.7)	4 (2.3)	1.00

AS = artesunate; AQ = amodiaquine; AL = artemether-lumefantrine; CD = chlorproguanil-dapsone

* *exact P values*

### G6PD deficient children

There was no statistically significant differences in the parasitological or clinical failure rates by day 14 or day 28 between the AS+AQ and AL groups (Table 6). The drop in mean Hb by day 3 post treatment and the recovery in Hb by day 28 were comparable between AS+AQ and AL groups and there was no apparent difference in the distribution of Hb concentrations following treatment between the G6PD normal and deficient children (data not shown).

**Table 6:** Treatment outcomes in G6PD deficient children (Per protocol analysis)

Outcomes	AS+AQ [N = 44]	AL [N = 42]	P*
	n (%)	n (%)	
Early treatment failure	1 (2.3)	0 (0.0)	0.3
Late treatment failure	1 (2.3)	2 (4.8)	0.5
Parasitological or clinical failure by day 14	3 (6.8)	2 (4.8)	0.7
Parasitological or clinical failure by day 28 (PCR uncorrected)	11 (25.0)	11 (26.2)	0.9
Parasitological or clinical failure by day 28 (PCR corrected)	6/43 (14.0)	4/42 (9.5)	0.5
Missing PCR by day 28 (excluded)	1 (2.3)	0	0.3

AS = artesunate; AQ = amodiaquine; AL = artemether-lumefantrine; CD = chlorproguanil-dapsone

* *exact P values*

## Discussion

In our study population, the parasitological and clinical failure at day 28 was lower in the AS+AQ group than in AL group. Our PCR corrected parasitological failure rate at day 28 is higher than that previously reported from Ghana[8] for AS+AQ (6.6% vs 2.5%) and remarkably higher for AL (13.8% vs 0%). The reason for the substantially higher parasitological failure at day 28 for AL compared to the results of a previous study in Ghana[8] and elsewhere[9] is unclear. Dietary differences between our population and the previously studied populations is a possible explanation for this difference; poor absorption of AL leading to a higher parasitological failure compared to that reported from elsewhere. We did not follow the recommended practice of administering AL with a fatty diet in a clinical trial; instead we only advised caretakers to give fatty food at the time of drug administration. This was a pragmatic decision because in routine health care setting one can only advise a caretaker to administer AL with a fatty meal. Thus, the observed higher treatment failure of AL observed in our study compared with others may have been due to the lack of intake of fat along with AL, as is likely to happen frequently in routine clinical practice.

Although there was a significant difference in the parasitological failure rate (both recrudescence and re-infections) at day 28 between AL and AS+AQ, there was no difference in the clinical failure between the two groups, perhaps because both combinations contain a fast acting artemisinin.

The parasitological or clinical failure of AS+CD by day 14 was 8% and by day 28 (PCR corrected) was 13.8% in our study population. There are no other published studies of efficacy of AS+CD with which to compare these results. An earlier multicountry trial of CD monotherapy showed a parasitological or clinical failure rate of 5% by day 14 in Nigeria.[10] Our study suggests that adding AS to CD may have little value in West Africa given that the PCR corrected failure rate by day 28 has already reached 13.8%. Surprisingly, the PCR corrected failure rate by day 28 for AL was also 13.8% and this suggests that introducing AL as a replacement for AS+AQ needs to considered with caution. More evidence on the efficacy of AL in Ghana is needed urgently.

In the AL group, all children who had gametocytes on day 0 (n = 11) had cleared gametocytes by day 3 post treatment; gametocytes appeared in the blood of two children between day 14 and 28. In children in the AS+AQ group who had gametocytes on day 0 (n = 10) only one child had gametocytes on day 3 and 7. Two more children developed gametocytaemia by day 7 but all children cleared gametocytes by day 14. In children in the AS+CD group who had gametocytes on day 0 (n = 16) 4 children had gametocytes on day 3 and one child had gametocytes on day 7 Gametocytes appeared in a further child by day 7 but all children had cleared gametocytes by day 14; two children had developed gametocytes between day 14 an 28. It appears that all three ACTs tested in this study are effective at clearing gametocytes and this property of ACTs should have an effect on the transmission of malaria.

A slight reduction in the Hb concentration occured during the early post treatment period in all three treatment groups. Although differences were not remarkable, the recovery of Hb was slowest following treatment with AS+CD. There was no difference in the risk of haemolysis between the three ACTs in G6PD normal children. However, we cannot comment about the potential risk of haemolysis associated with AS+CD in an unselected population because G6PD deficient children were not included in the AS+CD arm. Results from recently completed multicentre trials indicate that coformulated AS+CD can cause serious haemolysis in children who are G6PD deficient so further development of this drug combination has been halted.

Our study results supports the current policy of AS+AQ as first line treatment for uncomplicated malaria in Ghana. However, the slightly higher incidence of adverse events, particularly vomiting and body pain, associated with use of AS+AQ is a concern. If these adverse events are common in adults as well then they might reduce adherence to a full course of the treatment. There were some reports of adverse events of AQ in the Ghanaian mass media and this has lead to the general public being reluctant to accept AS+AQ combination therapy. Although the overall incidence of adverse events related to AS+AQ group is reasonable, in order to achieve a good compliance an appropriate communication strategy is needed in addition to the introduction of the new fixed combinations of artesunate plus amodiaquine that are currently being developed.

We conclude that AS+AQ is an appropriate first line treatment for uncomplicated malaria in Ghana and possibly in neighbouring countries in West Africa. However, the deployment of AS+AQ should be linked to a clear information strategy regarding the potential mild adverse events for health care providers and the general public. The efficacy and safety of AL when given in the context of routine care need to be further studied in Ghana and elsewhere in West Africa.

## Supporting Information

Protocol S1 Trial Protocol.
(0.29 MB DOC)

Checklist S1 CONSORT Checklist.
(0.06 MB DOC)
