# Supplementary material for: An Open Label, Randomised Trial of Artesunate+Amodiaquine, Artesunate+Chlorproguanil-Dapsone and Artemether-Lumefantrine for the Treatment of Uncomplicated Malaria
Source: PLoS One. 2008 Jun 25;3(6):e2530. doi: 10.1371/journal.pone.0002530 (PMC2430614; doi:10.1371/journal.pone.0002530)
Supplement: Protocol S1 — Trial Protocol. (0.29 MB DOC) [file pone.0002530.s001.doc]

**GATES MALARIA PARTNERSHIP**

***APPLICATION FORM - PROJECT GRANTS***

**TITLE OF PROPOSAL:** A non-inferiority, open-labelled, randomised trial of the efficacy

and safety of artesunate-amodiaquine, artemether-lumefantrine, and artesunate-lapdap for treatment of uncomplicated *P. falciparum* malaria among children in Ghana.

**PRINCIPAL INVESTIGATOR(S)**

Seth Owusu-Agyei, Brian Greenwood, John Gyapong, Daniel Chandramohan

**COLLABORATORS AND AFFILIATIONS**

K.P. Asante, MD, MPH; Kintampo Health Research Centre, GHS, Kintampo. Ghana

Kojo Koram, MD, PhD, Noguchi Medical Research Institute, UG. Legon. Ghana

Michael Wilson PhD: Noguchi Medical Research Institute, UG. Legon. Ghana

Martin Adjuik, MSc, Navrongo Health Research Centre, Navrongo. Ghana

Fred Binka, MD PhD; INDEPTH Network, Adendum Loop, Accra. Ghana

**PROPOSED STARTING DATE: September 2004 DURATION: 15 MONTHS**

# TOTAL BUDGET REQUESTED: £ 145,353

**NEW STAFF REQUIRED (if applicable) Not Applicable**

**Grade Number Required Proposed Location (office/lab)**

**PROFESSIONAL DETAILS OF PRINCIPAL INVESTIGATORS**

Dr Seth Owusu-Agyei

Director, Kintampo Health Research Centre

Health Research Unit, Ghana Health Service

P. O. Box 200. Kintampo. Ghana.

Tel 233 61 24145

Fax 233 61 24145

Email: Seth.Owusu-Agyei@ghana-khrc.org

Professor Brian Greenwood

Professor of Clinical Tropical Medicine

Department of Infectious and Tropical Diseases, LSHTM

Keppel St, London, WCIE 7HT

Tel. 44 20 7299 4707

Fax 44 20 7580 9075

Email. Brian.Greenwood@lshtm.ac.uk

Dr John Gyapong

Director, Health Research Unit

Ghana Health Service, Ghana

P. O. Box 184 Accra, Ghana

Tel: 233 21 681085

Fax 233 21 226739

Email: John.Gyapong@hru-ghs.org

Dr Daniel Chandramohan

Senior lecturer

Department of Infectious and Tropical Diseases, LSHTM

Keppel St London WCIE 7HT

Tel. 44 20 7927 2322

Fax 44 20 7580 9075

Email. Daniel.Chandramohan@lshtm.ac.uk

**SUMMARY**

Case management is the key strategy being employed in malaria control in most endemic areas of the world. *Plasmodium* *falciparum* malaria is unfortunately becoming resistant to commonly used and cheap antimalarial drugs such as chloroquine, amodiaquine, and sulfadoxine-pyrimethamine (SP). New and promising anti-malaria drugs need to be optimally assessed in sites with well-characterised malariometric indices in order to make the efficacious and safe ones available

Artemisinin-based combination chemotherapies have been documented to consistently produce faster relief of clinical symptoms and parasite clearance in uncomplicated *falciparum* malaria than any other currently used antimalarial drugs. We so far have artesunate-amodiaquine (AS-AQ) and artemether-lumefantrine (AR-LM) as the only two registered fixed-dose artemisinin combination chemotherapies produced at industrial scale, to good manufacturing practices and already used in Africa. Several African countries, including Ghana, are therefore either introducing AS-AQ or AR-LM as first-line antimalarials or evaluating the case for such a change. Clearly, a direct comparison of both the safety and efficacy profiles of the two combinations under different epidemiological conditions is urgently needed to guide informed decisions on the most appropriate antimalarial first-line treatment regimen within the framework of evidence-based health care and disease control programmes.

SP had been regarded as the potential drug of choice for intermittent, preventive treatment in infants (IPTi) in Ghana as treatment with SP is easy to administer; can be carried out under direct observed therapy and has a relatively longer half-life. SP has to be replaced, due to high parasitological and clinical failure rates from recent data generated in parts of Ghana. Lapdap has been found to clear parasites where SP has failed. An artesunate-lapdap combination therapy could be another option, i.e. if a fixed-dose formulation becomes available in the near future. Evidence for a suitable replacement antimalarial drug is crucial to consolidate the large-scale implementation of IPTi that is envisaged to play a major role in malaria control in Ghana.

We propose to evaluate the efficacy and safety of artesunate-amodiaquine combination therapy (AS-AQ), Artemether-lumefantrine, and artesunate-lapdap in an open-labelled, randomised, non-inferiority drug trial.

The study results will directly inform future decisions on first- and second-line treatments for uncomplicated *P. falciparum* malaria with respect to efficacy and safety in Ghana and also for their suitability as choice of antimalarial for an IPTi programme.

**RESEARCH PROPOSAL (Maximum 10 pages - font size 12)**

**Background to the study**

Case management of malaria is one of the key strategies being employed in most malaria endemic areas in the world. Because *Plasmodium* *falciparum* malaria is becoming resistant to commonly used and cheap antimalarial drugs such as chloroquine (CQ), amodiaquine (AQ), and sulfadoxine-pyrimethamine (SP), new and promising anti-malaria drugs need to be optimally assessed in sites with well-characterised malariometric indices in order to make the efficacious and safe ones available to affected individuals and avert deaths. A study funded by the GMP-LSHTM in the Kintampo district of Ghana is currently assessing the efficacy of SP as part of a comprehensive process of characterising the site into the patterns of seasonal dynamics of *P.* *falciparum* transmission, infection, and morbidity. The initial results from this study has demonstrated clearly, a high parasitological failure rate of 18% on day 14 of treatment, an indication that SP is no more suitable for use in an IPTi programme in Ghana. Prior to this initial trial results, SP, which is easy to administer under direct observed therapy and has a relatively longer half-life, was regarded as the potential drug of choice for intermittent, preventive treatment in infants (IPTi) in Ghana subject to the availability of supportive results on successful completion of current trials in northern and mid-Ghana. Evidence for a suitable replacement antimalarial drug that is efficacious, safe and easy to administer is crucial to consolidate the large-scale implementation of IPTi in malaria control since recent results that have shown that giving antimalarial three times in infancy (at the time of routine immunisation or at health visits) protect children from both clinical malaria and anaemia ^1, 2^.

ACTs have been found to be efficacious and safe, producing rapid clearance of parasites and malaria symptoms; they are very well tolerated, and are associated with very good compliance ^3, 4, 5^. No substantial evidence for artemisinin-induced toxicity ^6,7^.

Lapdap is a newly registered, relatively cheap antimalarial with short half-life and has been found to be highly efficacious in strict trial conditions for treatment of acute uncomplicated *falciparum* infections in endemic sites in Africa^8^. Despite the rapid clearance of lapdap, children treated with this drug did not have higher incidence of malaria episodes than those treated with SP ^9,10^ though haematological adverse effects have been documented to be more common with lapdap than with SP ^11^.

At present, the fixed-dose combination regimens of artesunate-amodiaquine (AS-AQ) and artemether-lumefantrine (AR-LM) are the only two registered artemisinin combination chemotherapies produced at industrial scale, to good manufacturing practices and already used in Africa. Several African countries are either introducing AS-AQ or AR-LM as first-line antimalarials or evaluating the case for such a change. Clearly, a direct comparison of both the safety and efficacy profiles of the two combinations under different epidemiological conditions is urgently needed to guide informed decisions on the most appropriate antimalarial first-line treatment regimen within the framework of evidence-based health care and disease control programmes.

Ghana, has just changed its antimalarial drug policy to artesunate-amodiaquine combination therapy (AS-AQ) as first line drug in order to reflect the new drive towards artemisinin-based combination treatment (ACT) that have been documented to have excellent tolerability, high cure rates, and to reduce gametocyte carriage in the treatment of uncomplicated *P. falciparum* malaria ^5, 12, 13^. The selection of this new ACT has been driven partly by cost of treatment and also unlikely threat of resistance, but a critical look at the safety and efficacy of ACTs in Ghana has yet to be done. An important first step is to evaluate this new drug at baseline, and develop this as a sentinel surveillance system in order to inform policy-makers in Ghana about its safety and efficacy profile over time.

We propose to evaluate the efficacy and safety of artesunate-amodiaquine combination therapy (AS-AQ), Artemether-lumefantrine (Coartem), and Artesunate-lapdap) in a drug non-inferiority study.

As we assess the suitability of AS-AQ as first line antimalarial in Ghana, this study will also assess the suitability of Coartem (AR-LM) and or artesunate-lapdap (AS-LP) as replacement drugs for AS-AQ and also for their suitability as choice of antimalarial for an IPTi programme. The study results will directly inform future decisions on first- and second-line treatments for uncomplicated *P. falciparum* malaria with respect to efficacy and safety in the population at highest risk ^11^.

# Objectives

**Primary objective**:

- To evaluate the efficacy of artesunate-amodiaquine versus artemether-lumefantrine, versus artesunate-lapdap in the treatment of children 6 months to ten years with uncomplicated *falciparum* malaria at paediatric outpatient clinic in the Kintampo hospital.

**Secondary objectives:**

- To evaluate the safety of artesunate-amodiaquine versus artemether-lumefantrine, versus artesunate-lapdap in the treatment of children 6 months to ten years with uncomplicated *falciparum* malaria at paediatric outpatient clinic in the Kintampo hospital.
- To develop and strengthen competitive and sustainable clinical research capacities for GCP-compliant clinical trials evaluating new antimalarial drugs in an endemic site in Africa.
- To use the safety and efficacy data and the ease of application to select the best one of the three to be used in an IPTi programme in Ghana.

Of particular interest in this investigation are:

**a). Efficacy:** Clinical and Parasitological response to treatment, monitored up to

day 28 following treatment as per current WHO protocols**^5^**, will be determined . Gametocyte production will be monitored carefully; recrudescence and re-

infection will be differentiated by PCR genotyping.

**b). Safety:** Safety and adverse events will be assessed including potential

effects on haematologic, renal, and hepatic function. Children tested and

documented with known glucose-6-phosphate dehydrogenase (G6PD)

deficiency will not be treated with Chlorproguanil-dapsone (CL-DP) as recent

literature supports CL-DP is contraindicated in patients with known G6PD

deficiency; these patients are likely to be susceptible to the haemolytic effects

such as reported drop in haemoglobin following treatment due to the dapsone

component of this drug. Rigorous checks will be on daily basis during the

treatment phase of the study to pick up any children at risk and put in

remedy.

**c). Molecular causes of efficacy:** The potential for chlorproguanil-dapsone (CL-DP), to select for resistance will be monitored by studying dehydrofolate reductase (dhfr) and dihydropteroate synthase (dhps) mutations.

**Study design and methods**

**Study site:** This study will be conducted in Kintampo district in the middle belt of Ghana where the investigators in the Kintampo Health Research Centre are located. Kintampo Health Research Centre (KHRC), one of three research centres under the Health Research Unit (HRU) of the Ghana Health Service, Ghana, lies within the forest-savannah, transitional ecological zone in the Brong Ahafo Region of Ghana. It has an area of 7,162 km^2^ and a population of approximately 160,000 people living in roughly 16,000 houses in 139 villages. KHRC is implementing a demographic surveillance system that gathers routine data on births, deaths and migrations in the Kintampo District. The Centre has fieldwork offices, laboratory, high-volume on-site data management computer centre, training rooms, library, and stores. KHRC has a core of experienced research staff, a cadre of trained field staff, with facilities for recruiting and training additional staff for projects as required. Kintampo district has 4 rural clinics, 3 health centres, 1 urban clinic and 1 district hospital located next to KHRC, and involved in joined research activities.

Communities engage in farming of predominantly maize and yam and to some extent, livestock. The area has a mean monthly temperature range of 18°C to 38°C. Rainfall averages 1250 mm per annum in 2 defined rainy seasons, occurring mainly between April and July, and between September to October when transmission by *Anopheles* peaks. Malaria is the leading cause of morbidity (52%) and mortality (32%) in the health facilities in the Kintampo district. Mortality resulting from anaemia constitutes 12% of deaths in the health facilities. Though members of the communities may be using one control method or another, no organised control activities or research have been put in place. There is no organised insecticide treated bednet marketing. Though the national policy on prompt appropriate treatment applies, treatment-seeking behaviour is purely the decision of the individual or members of the family, leading to delays in treatment or inappropriate treatment.

The laboratory has some standard devices, including a deep freezer, and two refrigerators for processing and storage of biological samples being collected on current projects. A laboratory technologist, two technicians, an assistant biochemist and research assistants are trained to cope with malaria-related examinations such as microscopy. Routine PCR and ELISA assays techniques will soon be transferred from the London School of Hygiene & Tropical Medicine. It is expected that detailed chemistry and haematology equipment will be made available for our well-qualified staff to embark on this and other future studies.

Communication is good due to availability of a locally managed VSAT that ensures a 24-hour internet connectivity to KHRC.

Current research being conducted in the district attests to the fact that *falciparum* resistance to chloroquine is intense, and resistance to SP is unexpectedly high.

**Study design**

This is a randomised, open-labelled, non-inferiority drug trial. At the Kintampo district hospital, 510 paediatric outpatients (refer sample size calculation) with uncomplicated *P. falciparum* malaria and aged between 6 months and 10 years will be recruited and randomly assigned to one of the three study arms: (i) Artesunate-Amodiaquine (AS-AQ), (ii) Artemether-Lumefantrine (AR-LM), or (iii) Artesunate-lapdap (AS-LP). The classification of clinical and parasitological responses will follow the relevant WHO protocol for areas of intense transmission ^14^. Follow-up, however, will be extended beyond day 14 up to day 28 to increase the sensitivity of the *in vivo* test. PCR-based genotyping comparing pairs of parasite isolates from day 0 and day of asexual parasite reappearance will be used to distinguish between recrudescence and re-infection.

### Primary endpoint

• Parasitological cure rates by day 14.

### Secondary endpoints

• Parasitological cure rate by day 28

• Clinical cure rates by days 14 and 28, as well as fever clearance times.

• Incidence rates of adverse events, including haematological and biochemical

evidence of drug-induced toxicity

• Carrier rates of sexual parasite stages at days 7, 14 and 28

### Randomisation

Pre-defined inclusion and exclusion criteria will be applied prior to recruiting. Allocation of qualified study participants to one of the three study arms [Artesunate-Amodiaquine (AS-AQ), Artemether-Lumefantrine (AR-LM), or Artesunate-lapdap (AS-LP)] will be done individually using a computer-generated randomisation list with a block size of 15. Sequentially numbered sealed envelopes matching the chronological study number of participants will contain the information on treatment allocation from the randomisation list. This will ensure the concealment of treatment allocation until administering the study medications.

**Candidate interventions**

Eligible patients will be randomly assigned to receive one of the following three study medications under direct observation:

Artesunate-Amodiaquine: about 4 mg/kg body weight of artesunate p.o. and

about 10 mg/kg body weight of amodiaquine at hours

0, 24, and 48 (3 doses)

Artemether-Lumefantrine: about 2 mg/kg body weight of artemether p.o. and

about 12 mg/kg body weight of lumefantrine p.o. at

hours 0, 8, 24, 36, 48, and 60 (6 doses)

Artesunate-Lapdap: about 4 mg/kg body weight of artesunate p.o.,

about 2 mg/kg body weight of Chlorproguanil p.o. and 2.5 mg/kg body weight of Dapsone p.o. as single

dose at hours 0, 24, and 48 (3 doses)

Fixed doses for a given range of body weights will be used for treatment of the children enrolled in this study. This will be finalised prior to initiation of the study.

*Treatment-related Standard Operating Procedure*

Patients who qualify to be recruited into the study will be enrolled at the OPD of the Kintampo district hospital. All treatments will be carried out at the hospital under direct observation. This means that the patients and their caretakers will be compensated for their travel expenses to and from the hospital and time in the hospital. Patients with treatment failure and those developing severe malaria will be referred to the Kintampo district hospital for quinine treatment. Though the study will be an open-labelled one, the field staff involved in making follow-up observations or in undertaking laboratory measurements will be blinded from knowing which group a particular patient belonged to.

All drug administrations will be supervised and administered according to a Standard

Operating Procedure; full re-dosing will be performed, if vomiting occurs within 30 minutes. Every drug administration will be recorded on a drug accountability form. If a patient is unable to tolerate the trial medication and treatment is discontinued, and reasons recorded on the case record form as an adverse event and rescue medication must be initiated.

*Concomitant Treatment*

Patients will be treated for malarial symptoms and adverse events during the study period by standard treatment. Drugs likely to have an effect on parasitaemia will not be permitted during the study, if clinically necessary this is a reason for exclusion

*Clinical Trial Material*

**1. Artesunate-amodiaquine (AS-AQ)**

Artesunate-amodiaquine will be supplied as combination blister pack (Arsumac®) containing tablets of 50 mg artesunate plus tablets of 200 mg amodiaquine hydrochloride (153 mg amodiaquine base) on the same blister pack (thermoformed aluminium/PVC packaging). This co-packaged formulation of artesunate-amodiaquine is produced to Good Manufacturing Practices (GMP) and will be supplied by Sanofi-Synthélabo, a drug company listed by WHO as one of the two suppliers of artemisinin-based antimalarial combination of acceptable quality. The development of the combination blister pack of artesunate-amodiaquine has support from TDR/WHO^15^. Contact for supply will be made as soon as the fate of this proposal is determined.

Data on the safety and efficacy of artesunate-amodiaquine for the treatment of uncomplicated *P. falciparum* malaria came from a large multicentre randomised controlled trial ^11^. The haematological and hepatotoxic adverse effects of amodiaquine including fatal agranulocytosis are conclusively documented but these effects have been attributed to its use in chemoprophylaxis but not in chemotherapy^16^. However, a recent multicentre study found evidence of neutropenia occurring in 4 % of paediatric outpatients with uncomplicated *P. falciparum* malaria treated with amodiaquine either alone or in combination with artesunate in one study site^7^. In addition, a case of acute asymptomatic hepatitis was recently reported in a volunteer receiving artesunate-amodiaquine^17^.

**2. Artemether-lumefantrine (AM-LM)**

Novartis will supply artemether-lumefantrine as fixed-dose combination tablets (Coartem®) containing 20 mg of artemether and 120 mg of lumefantrine. Contact for supply will be made as soon as the fate of this proposal is determined. This fixed-dose co-formulation of artemether-lumefantrine is currently registered for the treatment of children aged =12 years and weighing at least 35 kg. The proposal intends to capitalize on available clinical data in paediatric patient populations and use the already registered co-formulation of artemether-lumefantrine. This will include the paediatric co-formulation under investigation currently with support by Medicines for Malaria Venture^18^.

Adverse effects reported through use of artemether-lumefantrine include: headache, dizziness, sleep disorders, palpitation, GI disorders, skin disorders, cough, fatigue, arthrialgia, and myalgia^19^.

**3. Artesunate-lapdap**

Chlorproguanil-Dapsone will be bought locally in Ghana as it is licensed in the country and sold in the main pharmacies. Chlorproguanil has half-life of 20 hours and Dapsone 17-33 hours. Drug combination is contraindicated for patients with liver failure, history of G6PD deficiency or intravascular haemolysis. Adverse effects may include: fever, convulsions, anaemia, GI effects, mouth ulcers, anorexia, neuropathy, allergic dermatitis^17^. Preliminary data from Malawi shows that the Artesunate-lapdap is safe (unpublished data). The co-formulated (AS-LP) will not be ready within the next couple of years, so we will use appropriate, but separate (unfixed) artesunate and lapdap doses with lapdap given to only G6PD normal children.

### Alternative therapy for treatment failures

In the event of withdrawal during the treatment phase, administration of the study drug will be discontinued. If the patient is still parasitaemic, the first line antimalarial, which is artesunate-amodiaquine antimalarial will be given to the patient to complete the treatment at home. Patients with treatment failure before day 14 or between days 14 and 28 will be treated with quinine, the second line drug. Those requiring parenteral treatment will be given iv-quinine and appropriate supportive treatment. These patients will be referred to the Kintampo district hospital for admission and further care.

### Accountability procedures for the investigational drugs

The Principal Investigators will ensure that all clinical supplies are properly received, stored and documented. The Principal Investigators will give the study medications to the dispensing staff. The dispensing staff will then account for all used and unused drug supplies. A product accountability list will be recorded and kept in the investigator’s file.

The remaining medication will be kept in the original package and stored for clinical monitoring and drug accounting purposes.

All investigational products will be used only in accordance with the study protocol and separately provided Standard Operational Procedures.

**Selection and Withdrawal of Subjects**

### Inclusion criteria

• Male and female outpatients aged 6months to 10 years

• Body weight >5 kg

• Uncomplicated *P. falciparum* malaria

• Mono-infection with *P. falciparum* with an asexual parasite density between 1,000 to

200,000 parasites/µl

• Haemoglobin ≥9.0 g/dL

• Axillary temperature ≥37.5^o^C or history of fever in preceding 24 hr

• Ability to tolerate oral therapy

• Informed consent by the parents of the subject; Oral agreement of older children

• Residence in study area, i.e. possession of Kintampo Demographic Surveillance

System number.

### Exclusion criteria

• Haemoglobin <9.0 g/dL

• Haematocrit <25 %

• Leucocyte count: >15,000/µL

• Mixed plasmodial infection

• Danger signs (unable to drink; repeated vomiting; recent history of convulsions;

lethargic or unconscious state; unable to stand up or to sit) and signs of severe

malaria according as defined by WHO^20^

• Any other severe underlying disease (cardiac, renal, hepatic diseases, malnutrition,

known HIV infection)

• Concomitant disease masking assessment of response, e.g. known or suspected

hearing impairments

• History of allergy or intolerance against study medications

• Previous participation in this clinical trial

### Trial procedures and study flow

A general physical examination will be performed on enrolment into the study. Subsequently, brief examinations will be conducted daily for the first week and once weekly thereafter to assess general physical status, hepatic and splenic enlargement.

The study physician, assisted by a clinical nurse will closely monitor all patients during the treatment phase (day 0-2). Thereafter, patients will return for outpatient follow-up on days 3, 7, 14, 28 for safety and efficacy assessments including parasitological, haematological and biochemical evaluations. The study physician will be made to examine each patient and at all the contacts. Blood blots will also be collected on filter paper on days of blood sample collection for genotyping. The procedures are detailed out in Table 1 below.

Those patients who fail to attend to outpatient follow-up will be located and transported to the hospital for the scheduled examinations or ascertain the reason for non-attendance on the follow-up visit.

Table 1: Schematic diagram of trial procedures

|  | **Day(s)** | | | | | | | |
| --- | --- | --- | --- | --- | --- | --- | --- | --- |
|  | **0** | **1** | **2** | **3** | **7** | **14** | **21** | **28** |
| Physical exam, medical history | **X** | **X** | **X** | **X** | **X** | **X** | **X** | **X** |
| Clinical examination | **X** | **X** |  | **X** | **X** | **X** | **X** | **X** |
| Temperature, Pulse, Blood pressure | **X** | **X** | **X** | **X** | **X** | **X** |  | **X** |
| Treatment | **X** | **X** | **X** |  |  |  |  |  |
| Parasitaemia, Gametocyte | **X** |  |  | **X** | **X** | **X** |  | **X** |
| Filter paper blot for PCR | **X** |  |  | **X** | **X** | **X** |  | **X** |
| Haematology | **X** | **X** | **X** | **X** | **X** |  |  | **X** |
| Differential blood count | **X** |  |  | **X** | **X** |  |  | **X** |
| Liver function | **X** |  | **X** |  | **X** |  |  | **X** |
| Renal function | **X** |  | **X** |  | **X** |  |  | **X** |
| Adverse events documentation | **X** | **X** | **X** | **X** | **X** | **X** | **X** | **X** |
| Other | **X** | **X** | **X** | **X** | **X** | **X** | **X** | **X** |

**Parasitological examinations:** Thin and thick smears slides prepared from all volunteers will be dried, the thin films fixed with methanol and both stained with Giemsa. These will be examined for parasites. The microscopist who examines each slide could declare it negative, only after 200 oil immersion fields of the thick films has been examined using x10 eyepieces and x100 oil immersion lens binocular microscope and no malaria parasite is seen. The number of parasites per micro-litre approximately equals 40 times the number of parasites counted per 200 leukocytes. This assumes a white blood cell count of 8,000 WBC/micro-litre. This information will be entered on the participant’s data form and in the computer database.

**Re-Infection** will be differentiated from recrudescence by comparing genotype patterns obtained on the day of re-appearing parasitaemia with that of the day 0 isolate. Geno­typing will be achieved by PCR-amplification of *P. falciparum msp2*.

***dhfr/dhps* genotypes.** All day 0 isolates will be genotyped for pf*dhfr/pfdhps* by PCR-RFLP and molecular typing will be undertaken in any patient in the lapdap group who fails treatment. Analysis of the day 0 samples will provide important information on the prevalence of molecular markers of SP resistance; this is important as it is currently proposed to employ lapdap in a large IPT trial in the study area.

**Pre-treatment antimalarial drug levels** will be assessed by ELISA assays on chloroquine, amodiaquine, sulfadoxine and pyrimethamine.

**Monitoring for side effects:** All children will be clinically examined during scheduled and unscheduled visits and a clinical history will be obtained. Adverse events (AEs) will be considered as any clinically significant medical condition occurring during follow-up. AEs will be grouped into non-severe and severe (SAE) and the probability of association with antimalarial treatment will be estimated. SAEs will be defined by the occurrence of an AE that results in: life-threatening condition, death, hospitalisation, disability, or any condition requiring intervention to prevent these. All children will be followed up until the AE or SAE has resolved, can be explained otherwise, or the particular child has been lost to follow-up. Emphasis will be put on skin effects, haematology (Hb, differential blood count), liver function (ALT, AST, bilirubin), and renal function (creatinine, urea). Since lapdap is contraindicated for G6PD deficients, all the children will be tested for their G6PD status. The G6PD deficient children will thereafter be excluded from taking lapdap due to their status; the other study drugs will be administered to all other children randomised to receive it without discrimination. To avoid any acute haemolysis in this study, periodic haemoglobin will be measured by a HaemoCue photometer. Differential blood counts will be performed on May-Gruenwald-stained thin blood films. Liver and kidney markers will be assessed photometrically (Reflotron, Roche).

**Monitoring for compliance**

Non-involved scientists in the Kintampo Health Research Centre (KHRC) will monitor the clinical trial for compliance within a GCP framework. This will include on-site monitoring to detect inconsistencies and non-compliance using trial documentation and available data at any stage. A strong emphasis will be placed on detailed, efficient and timely adverse event reporting and absolute adherence to the study protocol with respect to the specific safety requirements in study participants who will be re-treated with the same study medication in case of (i) treatment failure between days 14 and 28 or (ii) uncomplicated *P. falciparum* malaria on day 28. Reports will be kept as Monitoring Logs at the study site. Monitoring will be integrated into the development of capacity strengthening, training and education training module for other scientists in the centre who are yet to trained in GCP-compliant clinical research.

###### Outcome measures. Primary endpoints are parasitaemia on day 28, classification of out­come into ACPR, ETF, LCTF, and LPTF, and AEs. Analysis will be *per protocol* and by intention-to-treat, as applicable.

### Trial Stopping Rules

All serious adverse events will be reported to the local safety monitor within 24 hours, and the local safety monitor in consultation with the chair of the DSMB will have the power to suspend the study while the investigation of the link between the adverse event and the trial drug is being established.

An interim analysis of changes or/and incidence rates of haematological, biochemical parameters etc. etc. of drug-induced toxicity will be performed after completion of enrolment of 50% of patients in order to prevent further exposure of unjustifiably large numbers of patients to such risks.

A fully competent Data Safety & Monitoring Board (DSMB) will be set up for the trial. This study will try to meet as many as possible of the requirements of GCP.

**Capacity development objectives in the project**

There are on site 3 epidemiologists, one trained to the PhD level; four clinical research fellows, with postgraduate training in Public Health; a demographer and a senior data manager as the core group. Most senior officers have undergone GCP training. Within the proposed project, two clinicians and two laboratory technicians/technologists are to be trained or re-trained in GCP and GLP, respectively. Training in internal monitoring is desirable as well as in laboratory quality assessment and quality check. Laboratory upgrading includes equipment and related training to measure liver and kidney function markers. The junior research associates will be involved in the conduct of the trial under supervision of the PI. Networking, staff exchange and participation in training units on "Assessing antimalarial efficacy in African settings" is desired in Kintampo Health Research Centre (KHRC).

The Computer Centre is responsible for all the data processing in KHRC. The Computer Centre processes around 30,000 data capture forms each week as part of current ongoing research activities in the centre. To ensure data quality, all forms are checked for completeness, and are double entered on the computer. The Centre has had to develop a rigorous data management system using Visual FoxProTM and Access in order to ensure data quality. As a result KHRC has developed a strong reputation for running complex, large-scale clinical trials. Additional capacity building is envisaged for our new data managers in handling clinical trials data.

**Sample size:**

The following assumptions are made for estimating the required sample size of 510 children in the three arms of the study:

- The efficacy of Artemether-Lumefantrine (AR-LM) is 95%^21^. We will therefore use an efficacy of 95% in this arm of the study.
- The decrease in efficacy for Artesunate-Amodiaquine (AS-AQ) and Artesunate-lapdap (AS-LP) will not be more than 10% of the efficacy of AR-LM. Thus the expected efficacy for AS-AQ & AS-LP will be 86%(90%of 95%)
- Loss to follow-up at 28 days post-treatment is put at ≤ 5%.
- Maximum precision of 10% and power 80%.

# Table 2: Sample size calculation

**_____________________________________________________________________** Anticipated population proportion (P), *Confidence level: 95%*

*d* 0.05 0.10 0.15 0.20 0.25 0.30

0.05 73 138 196 246 288 323

0.10 18 49 61 72 81 87

_____________________________________________________________________________

The study will thus require 170 per arm; a total sample size of 510 children will have 80% power and 90% significance to detect a 10% difference in the efficacy between AS-AQ, and AS-LP and that of AR-LM.

**(d) Time-table and milestones for achieving objectives**

The proposed study will have a duration of 1.5 years. Recruitment is expected to start in September 2004.

|  | **Year 1 (2004)** | | | | **Year 2 (2005)** | | | |
| --- | --- | --- | --- | --- | --- | --- | --- | --- |
|  | **1^st^ quarter** | **2^nd^ quarter** | **3^rd^ quarter** | **4^th^**  **quarter** | **1^st^ quarter** | **2^nd^ quarter** | **3^rd^ quarter** | **4^th^**  **quarter** |
| Proposal development |  | X | X |  |  |  |  |  |
| Preparations for implementation |  |  | X | X |  |  |  |  |
| Recruitment |  |  |  | X | X | X | X |  |
| Follow-Up |  |  |  | X | X | X | X |  |
| *dhfr/dhps* & *msp1/msp2* typing |  |  |  |  | X | X | X | X |
| ELISAs |  |  |  |  | X | X | X |  |
| Statistical analysis |  |  |  |  |  |  | X | X |
| Report write up, publication |  |  |  |  |  |  | X | X |

## References

1. Schellenberg D, Menendez C, Kahigwa E, Aponte J, Vidal J, Tanner M, Mshinda H, Alonso P. Intermittent treatment for malaria & anaemia control at time of routine vaccinations in Tanzanian infants: a randomised, placebo-controlled trial. Lancet 2001 May 12;357(9267):1471-7.

1. Massaga JJ, Kitua AY, Lemnge MM, Akida JA, Malle LN, Ronn AM, Theander TG, Bygbjerg IC. Effect of intermittent treatment with amodiaquine on anaemia and malarial fevers in

infants in Tanzania: a randomised placebo-controlled trial. Lancet. 2003 May 31;361(9372):1853-60.

1. White NJ, Olliaro P, 1998. Artemisinin and derivatives in the treatment of uncomplicated malaria. *Med Trop (Mars) 58*: 54-6.

4. Olliaro PL, Taylor WR, 2003. Antimalarial compounds: from bench to bedside. J. Exp Biol 206: 3753-9.

5. Adjuik M, Babiker A, Garner P, Olliaro P, Taylor W, White N, 2004. Artesunate combinations for treatment of malaria: meta-analysis. *Lancet 363*: 9-17.

6. Van Vugt M, Angus BJ, Price RN, Mann C, Simpson JA, Poletto C, Htoo SE, Looareesuwan S, White NJ, Nosten F, 2000. A case-control auditory evaluation of patients treated with artemisinin derivatives for multidrug-resistant P. falciparum malaria. *Am J Trop Med Hyg 62*: 65-9.

1. Hien TT, Turner GD, Mai NT, Phu NH, Bethell D, Blakemore WF, Cavanagh JB, Dayan A, Medana I, Weller RO, Day NP, White NJ, 2003. Neuropathological assessment of artemether-treated severe malaria. *Lancet 362*: 295-6.
2. Lang T, Greenwood B. The development of Lapdap, an affordable new treatment for malaria. Lancet Infect Dis. 2003 Mar;3(3):162-8.
3. Sulo J, Chimpeni P, Hatcher J, Kublin JG, Plowe CV, Molyneux ME, Marsh K, Taylor TE, Watkins WM, Winstanley PA. Chlorproguanil-dapsone versus sulfadoxine-pyrimethamine for sequential episodes of uncomplicated falciparum malaria in Kenya and Malawi: a randomised clinical trial. Lancet. 2002 Oct 12;360(9340):1136-43.
4. Mutabingwa T, Nzila A, Mberu E, Nduati E, Winstanley P, Hills E, Watkins W. Chlorproguanil-dapsone for treatment of drug-resistant falciparum malaria in Tanzania: Lancet. 2001 Oct 13;358(9289):1218-23
5. Alloueche A, Bailey W, Barton S, Bwika J, Chimpeni P, Falade CO, Fehintola FA, Horton J,

Jaffar S, Kanyok T, Kremsner PG, Kublin JG, Lang T, Missinou MA, Mkandala C, Oduola AM,

Premji Z, Robertson L, Sowunmi A, Ward SA, Winstanley PA. Comparison of chlorproguanil-

dapsone with sulfadoxine-pyrimethamine for the treatment of uncomplicated falciparum malaria

in young African children: double-blind randomised controlled trial. Lancet. 2004 Jun

5:363(9424): 1838-9.

12. von Seidlein L, Milligan P, Pinder M, Bojang K, Anyalebechi C, Gosling R, Coleman R, Ude JI, Sadiq A, Duraisingh M, Warhurst D, Alloueche A, Targett G, McAdam K, Greenwood B, Walraven G, Olliaro P, Doherty T, 2000. Efficacy of artesunate plus

pyrimethamine-sulphadoxine for uncomplicated malaria in Gambian children: a double-blind,

randomised, controlled trial. *Lancet 355*: 352-7.

13. Adjuik M, Agnamey P, Babiker A, Borrmann S, Brasseur P, Cisse M, Cobelens F, Diallo S,

Faucher JF, Garner P, Gikunda S, Kremsner PG, Krishna S, Lell B, Loolpapit M, Matsiegui PB,

Missinou MA, Mwanza J, Ntoumi F, Olliaro P, Osimbo P, Rezbach P, Some E, Taylor WR, 2002.

Amodiaquine-artesunate versus amodiaquine for uncomplicated Plasmodium falciparum malaria

in African children: a randomised, multicentre trial. *Lancet 359*: 1365-72.

1. WHO, 2003. Assessment and monitoring of antimalarial drug efficacy for the treatment of

uncomplicated falciparum malaria.

15. WHO: Procurement, Quality and Sourcing Project: Access to Artemisinin-based Combination Antimalarial - Drugs, of acceptable quality. Second edition, Geneva 26 April 2004).

1. Olliaro P, Nevill C, LeBras J, Ringwald P, Mussano P, Garner P, Brasseur P, 1996.

Systematic review of amodiaquine treatment in uncomplicated malaria. *Lancet 348*:

1196-201.

1. Orrell C, Taylor WR, Olliaro P, 2001. Acute asymptomatic hepatitis in a healthy normal volunteer

exposed to 2 oral doses of amodiaquine and artesunate. *Trans R Soc Trop Med Hyg 95*: 517-8.

18. Project title: Paediatric Coartem® initiative by MMV and the manufacturer (MMV: Development Projects. http://www.mmv.org)

1. WHO, 2001: The use of antimalarial drugs. Report of a WHO informal consultation.

WHO/CDS/RBM/2001.33. Nov 13-17, 2000.

1. Severe falciparum malaria. World Health Organization, Communicable Diseases Cluster

(2000).. *Trans R Soc Trop Med Hyg 94 Suppl 1*: S1-90.

1. Lefevre G, Looareesuwan S, Treeprasertsuk S, Krudsood S, Silachamroon U, Gathmann I, Mull

R, Bakshi R. A clinical and pharmacokinetic trial of six doses of artemether-lumefantrine for

multidrug-resistant Plasmodium falciparum malaria in Thailand. Am J Trop Med Hyg. 2001 May-

Jun;64(5-6):247-56.

**CONTRIBUTION TO THE OBJECTIVES OF THE GATES PARTNERSHIP**

**Indicate how this proposal meets the overall objectives of the Gates partnership including capacity development in a malaria endemic country (maximum 100 words)**

**CONTRIBUTION TO OBJECTIVES OF GATES PROGRAMME**

This study is a follow-up to the baseline studies of *falciparum* malaria in the Kintampo area that looked at the sensitivity of commonly used antimalarials drugs. Evaluation of these new anti-malarial drugs and drug combinations, will provide reliable, essential data on potential new antimalarials that could replace the old and failing antimalarials such and chloroquine and SP. Evidence for a suitable replacement antimalarial drug is crucial to consolidate the large-scale implementation of IPTi that is envisaged to play a major role in malaria control in Ghana. One of these combination drugs will be recommended following evaluation to be used for the IPTi malaria control in Ghana. The others will provide data that could be used for future antimalarial interventions in this area and beyond.

This proposal aims to develop and strengthen competitive and sustainable clinical research capacities within the Kintampo Health Research Centre as an excellent GCP-compliant clinical trials site able to evaluate new antimalarial drugs and potential future malaria vaccines in Africa. This capacity building measures will be achieved through a combined clinical and laboratory approach of the investigating team members in the proposed clinical trial.

# ANIMALS

Will animals be used in the project Yes/No **No**

If Yes, indicate: **Not Applicable**

**ETHICAL ISSUES**

**Discuss ethical aspects of the proposal.**

*Approval by the local IRB/IEC*

This study protocol will require approval from the relevant IRB/IEC of the study site and from all our collaborating institutions before it is initiated. The Principal Investigators will submit the study protocol with all necessary attachments and documentation to these authorities. A copy of the written approval from the local ethics committees will be distributed to all collaborating institutions involved in this piece of work.

*Subject Information and Informed Consent*

Each subject’s parent will receive written information about the study, which will also be explained in the presence of a witness if required. Sufficient time will be given to each subject’s parents to decide whether or not to participate in the study. Subjects will be given the opportunity to enquire about the details of the study and any question regarding the study will be answered. The Principal Investigators will ensure that the consent form has been signed and dated by the subject, physician and witness (if applicable). If required, an independent witness will attend the information session and sign and date the consent form prior to entry of the subject to the study. The Principal Investigators will ensure that the written information and the consent form are revised when an amendment to the clinical trial protocol is made and approved. A copy of the subject information and consent form as used will be given to the clinical monitor.

Before entering the study, each subject’s parent will give written informed consent. Consent will be verbal for anyone who is illiterate. In this situation, each subject’s parent will be required thumbprint on the informed consent form.

By signing this protocol, the Principal Investigators will assure that informed consent will be obtained and that the consent form and consent information to be used will be submitted to the Ethics Committee responsible for approving the conduct of the study. The recommendations guiding physicians in biomedical research involving human subjects issued by the World Medical Association Declaration of Helsinki will fully apply to this project.

*Trial-specific ethical considerations*

This trial aims to assess potential haematological, biochemical as well as yet unknown

drug-induced toxicities. The GCP-compliant development of such drugs for malaria in partnership with African research institutions to establish sustainable research capacity in Africa is the major objective of the GMP initiative and seen as a highly efficient investment aiming at the alleviation of poverty-related infectious diseases.

The Principal Investigators of this trial recognize the critical importance of accurate, efficient, timely and reliable safety assessments and related protection of study participants.

The proposed team of investigators have longstanding experiences in clinical trial methodologies. Cleary, rigorous monitoring will play a central role in this trial.

**Submission to LSHTM ethics committee** Y/N **Yes, but Not Yet**

**Submission to other ethics committee(s) (specify)** Y/N  **Yes, but Not Yet**

BUDGET (in £ sterling)

|  | **Year 1**  Sept.‘04-Aug.‘05 | | **Final 6 months**  Sept.’05-Jan.’06 | | **Total** | |
| --- | --- | --- | --- | --- | --- | --- |
| Capital equipment (List supplier, model and cost) | £ | | £ | | £ | |
| Clinical chemistry analyser (Randox Labs, UK; RX Daytona with ISE) | 7,000 | |  | | 7,000 | |
| Haematology analyser (ABX Diagnostics, France; Pentra series; ) | 5,000 | |  | | 5,000 | |
| Clinical Microscope, for urinalysis (Scientific Lab. Supplies, UK) | 1,613 | |  | | 1,613 | |
| Spectrophotometer, for G6PD/Chemisitry; Scientific Lab. Supplies, UK; Cecil model | 2,649 | |  | | 2,649 | |
| Water Bath, Scientific Lab. Supplies, UK; for G6PD/Chemistry | 1,000 | |  | | 1,000 | |
| Water distillation equipment (Scientific Lab. Supplies, Water still Hamilton) | 1,388 | |  | | 1,388 | |
| Weighing balance, Mettler Toledo model (Scientific Lab. Supplies, weighs: 0.1mg-220g) | 1,703 | |  | | 1,703 | |
|  | **20,353** | |  | | **20,353** | |
| Salaries List level and name if known) |  | |  | |  | |
| Clinician (1) for 100% time @ £ 500 each | 6,000 | | 2,500 | | 8,500 | |
| Assistant Research Officer, MSc (1) @ £ 400 | 7,200 | | 3,000 | | 10,200 | |
| Laboratory Technologists 2 @ 100% each for haematology, chemistry & microscopy @ £ 400 each | 9,600 | | 4,000 | | 13,600 | |
| Laboratory Assistants (2) to assist the Technologists £ 100 each | 2,400 | | 1,000 | | 3,400 | |
| Field workers, 6 @ 100% @ £100 each/month | 4,800 | | 2,000 | | 6,800 | |
| Data Manager, 1 @ £ 300 | 3,600 | | 1,500 | | 5,100 | |
| Data Entry Clerks 2 @ 50% of time each £100/mth | 1,200 | | 500 | | 1,700 | |
| Driver 1 @ 100% of time @ £100 | 1,200 | | 500 | | 1,700 | |
| Administrative support @ 30% time @£100 | 1,200 | | 500 | | 1,700 | |
| Financial Accounting support @ 30% time @ £100 | 1,200 | | 500 | | 1,700 | |
|  | **38,400** | | **16,000** | | **54,400** | |
| **Running costs** | |  | |  | |  |
| **- Laboratory** | |  | |  | |  |
| Reagents/Accessories for clinical chemistry analyses | | 10,000 | |  | | 10,000 |
| Reagents/Accessories for haematological analyses | | 5,000 | |  | | 5,000 |
| Microscope slides | | 1,000 | |  | | 1,000 |
| Reagents and accessories for microscopy work (lancets, Giemsa, gloves, filter paper, zip-locks, tubes, slide boxes, alcohol swabs, cotton wool etc.) | | 2,000 | | 500 | | 2,500 |
| PCR reagents and accessories & samples processing | | 3,000 | |  | | 3,000 |
| Shipping costs for items | | 3,000 | |  | | 3,000 |
|  | | **24,000** | | **500** | | **24,500** |
|  | |  | |  | |  |
| **- field expenses** | |  | |  | |  |
| Fuel and maintenance for 4WD | | 4,000 | | 1,000 | | 5,000 |
| Fuel and maintenance for motorbikes | | 2,000 | | 500 | | 2,500 |
| Insurance for vehicles (4WD and motorbikes) | | 1,000 | | 500 | | 1,500 |
| Support for running demographic surveillance | | 4,000 | |  | | 4,000 |
|  | | **11,000** | | **2,000** | | **13,000** |

|  | **Year 1**  Sept.‘04-Aug.‘05 | **Final 6 months**  Sept.’05-Jan.’06 | **Total** |
| --- | --- | --- | --- |
| **Running costs contd.** | £ | £ | £ |
| **- Clinical expenses** |  |  |  |
| Study Drugs | 4,000 |  | 4,000 |
| Other drugs (analgesics, antibiotics, haematinics etc) | 2,000 |  | 2,000 |
| Accessories for clinical use (Electronic thermometers, weighing scales, resuscitating kit, dressings etc) | 1,000 |  | 1,000 |
| T&T, hospital admission and feeding costs | 4,000 | 2000 | 6,000 |
|  | **11,000** | **2,000** | **13,000** |
| **- Communications** |  |  |  |
| Internet use, recurrent costs | 3,600 | 1,800 | 5,400 |
| Teleconference & Fax | 600 | 400 | 1,000 |
| Radio programme & Community liaison activities | 600 | 400 | 1,000 |
|  | **4,800** | **2,600** | **7,400** |
| **- Other (stationery/computing supplies)** |  |  |  |
| Stationery (duplicating paper, notebooks, pens, pencils, markers etc) | 1,000 | 500 | 1,500 |
| Printing of forms | 1,000 | 400 | 1,400 |
| Cartridges for printer | 500 | 200 | 700 |
|  | **2,500** | **1,100** | **3,600** |
| **Travel** |  |  |  |
| **- International** |  |  |  |
| Travel & Subsistence for OA to UK | 1,500 | 1,500 | 3,000 |
| Travel & Subsistence (DC scientific input & conduct of study) |  | 1,500 | 1,500 |
| Travel & Subsistence (BG scientific input & conduct of study) |  | 1,500 | 1,500 |
|  | **1,500** | **4,500** | **6,000** |
| **- Local** |  |  |  |
| Travel & Subsistence Kintampo- Accra for OA, KP (Noguchi, HRU meetings; organise equipment/items for the study); Accra-Kintampo for JG, WM, FB (to monitor progress of work and analyses workshop) | 2,000 | 1,000 | 3,000 |
|  | **2,000** | **1,000** | **3,000** |
| Miscellaneous |  |  |  |
|  |  |  |  |
|  |  |  |  |
| Summary |  |  |  |
| **- Capital** |  |  | **20,353** |
| **- Salaries** |  |  | **54,400** |
| **- Running costs** |  |  | **61,500** |
| **-Travel** |  |  | **9,000** |
| **TOTAL** |  |  | **145,253** |

**BUDGET JUSTIFICATION (Maximum 500 words)**

**Capital equipment:** All equipment requested in this budget took into consideration, the ones that were requested by PI for work in Kintampo Health Research Centre. All the previous will be transferred to this study to be funded by GMP. These include a vehicle 4WD, two motorbikes, two microscopes, PCR and ELISA equipment and two desk top computers.

We do not have any equipment for biochemical or haematological work, except old automated haemocues for haemoglobin determination. We have therefore included the costs of a clinical chemistry analyser and a haematology analyser with their accessories and reagents. We will need one additional microscope for urinalysis.

The water in Kintampo is hard and requires treatment. We currently arrange for de-ionised water from Noguchi, about 600 kilometres away. We will like to install a simple water distillation system in our lab in Kintampo Health Research Centre. The cost of very moderate distillation equipment has been requested.

**Personnel:** The request made takes into cognisance staff being trained in this clinical study as an essential core staff for future malaria intervention/clinical studies. It is expected that some members of the team could be further trained in disciplines currently lacking in the centre.

**Running cost:** The laboratory costs, field costs have been budgeted at levels to allow for smooth running of this project. Drug costs etc have been budgeted based on past experience, and not knowing exactly how much the pharmaceutical companies will charge us, but adequate to be able to buy some from any open markets.

The team will have to chase all defaulting participants. Detained participants will have to be fed, and cared for. Follow up reviews will require either picking participants from their homes, or paying their transportation into the research centre and back. With the availability of the project vehicle and motorbikes, the cost of fuel and routine servicing/maintenance of the 4WD vehicle has been budgeted for.

The money being requested for in this budget for internet use is to cater for 1/3 of the recurrent costs for the service.

**OTHER GRANTS HELD BY PI (List source of funding, amount and duration)**

1. Project Title: Patterns and characterisation of *Plasmodium falciparum* infections and morbidity in the Kintampo district in the middle belt of Ghana.

Source of funding: GMP-LSHTM.

Amount: £ 141,625

Duration: 1 year ending December 2004.

1. Project Title: A randomised, double-blind, placebo-controlled trial to evaluate the

impact of vitamin A supplementation on maternal mortality in Ghana

Source of funding: DFID, through LSHTM.

Amount: £ 4,000,000

Duration: 8 years ending May 2008.

# CO-APPLICATION

Has this grant been submitted elsewhere? Yes/No **No**

If Yes to whom **Not Applicable**

Status - under review

rejected

# CV OF PI AND MAIN COLLABORATORS (1 page)

| 1. Surname  OWUSU-AGYEI | Forename(s)  SETH | | Age  d.o.b. 25/12/1960 | |
| --- | --- | --- | --- | --- |
| 2. Degree, etc.  Subject  EPIDEMIOLOGY  MEDICAL PARASITOLOGY/ENTOMOLOGY  ZOOLOGY/CHEMISTRY | Degree  PhD  MSc  BSc | University  Swiss Tropical Institute  Liverpool School of Trop. Medicine  University of Ghana, Legon. | | Date  2001  1994  1986 |
| 3. Posts held (with dates: please identify tenure and source of funding of present post.  January 2003 to present: Postdoc Research Fellow, Gates Malaria Partnership, Dept. of Infectious  Tropical Diseases, LSHTM .  July 1 2002 to Present: Research Fellow, Public Health Intervention Research Unit, Dept. of  Epidemiology & Population Health, LSHTM, London  July 1 2002 to Present: Director, Kintampo Health Research Centre, Ministry of Health, Ghana.  2001 to Present: Epidemiologist, with speciality in malaria Epidemiology.  1999 to June 2002: Senior Health Research Scientist & Head of Biomedical Department,  Navrongo Health Research Centre, Ministry of Health, Ghana.  1992 – 1999: Research Officer, Navrongo Health Research Centre  1989 - 1992: Principal Research Asst, Ghana Vitamin A Supplementation Trials,  Navrongo.  1987 - 1989: Research Assistant, Epidemiology Unit. Noguchi Memorial Institute for  Medical Research. Legon. Ghana. | | | | |
| 4. Recent publications; also papers in press.  1. [Koram KA, Owusu-Agyei S, Fryauff DJ, Anto F, Atuguba F, Hodgson A, Hoffman SL, Nkrumah FK.](http://www.ncbi.nlm.nih.gov/entrez/query.fcgi?cmd=Retrieve&db=pubmed&dopt=Abstract&list_uids=12950665) Seasonal  profiles of malaria infection, anaemia, and bednet use among age groups and communities in northern Ghana.  Trop Med Int Health. 2003 Sep;8(9):793-802.   1. BR. Hale, **S Owusu-Agyei**, KA. Koram, DJ. Fryauff, M. Adjuik, AR. Oduro, WR Prescott, JK Baird, F. Nkrumah, ED. Franke, FN. Binka, J. Horton, SL. Hoffman: A Randomised, Double-blind, Placebo-controlled Dose-ranging Trial of Tafenoquine for Weekly Prophylaxis against *Plasmodium falciparum.* *Clin. Infect. Dis* **2003, March 1; 36(5): 541-9**. 2. J. Kevin Baird, **S. Owusu-Agyei**, Gregory C. Utz, Kwadwo Koram, Mazie J. Barcus, Stephen L. Hoffman, and Francis N. Nkrumah: Seasonal Malaria Attack rates in Infants and Young Children in northern Ghana. *Am. J. Trop. Med. Hyg.* **2002,** 66(3), 280-286. 3. **S Owusu-Agyei**, T. Smith, H-P. Beck, L. Amenga-Etego, I. Felger: Molecular Epidemiology of *Plasmodium falciparum* infections among asymptomatic inhabitants of a holoendemic malarious area in northern Ghana. *Trop. Med. & Int. Hlth.* **2002,** May 7(5), 421-428. 4. **S Owusu-Agyei**, Fred N. Binka, K. A. Koram, F. Anto, and M.Adjuik, F. Nkrumah and T. Smith: Does Radical Cure of Asymptomatic *P. falciparum* place Adults in Endemic Areas at Increased Risk of Recurrent Symptomatic Malaria? *Trop. Med. & Int. Hlth.* **2002,** July 7(7): 599-603. 5. **S Owusu-Agyei,** DJ Fryauff, D Chandramohan, KA Koram, FN Binka, FK Nkrumah, GC Utz, JK Baird and SL Hoffman. Characteristics of severe anaemia and its association with malaria in young children of the Kassena-Nankana District of Northern Ghana. Am J Trop Med Hyg **2002**, 67(4):371-7 6. **S Owusu-Agyei**, KA Koram, JK Baird, GC Utz, FN Binka, FK Nkrumah, DJ Fryauff, and SL Hoffman: Incidence of symptomatic and asymptomatic .*P. falciparum* infection following curative therapy in adult residents of northern Ghana: *Am. J. Trop. Med. Hyg.* **2001**, 65(3), 197-203. 7. K. A. Koram, **S Owusu-Agyei,** G. Utz, F. Binka, K. Baird, S. L. Hoffman, and F. K. Nkrumah: Severe Anaemia in Young Children after High and Low Malaria Transmission seasons in the Kassena-Nankana District of northern Ghana. *Am. J. Trop. Med. Hyg.* **2000**, 62(6), 670-674. | | | | |

| 1. Surname  GREENWOOD | | Forename(s)  BRIAN | | | Age 64  d.o.b. 11.11.1938 | |
| --- | --- | --- | --- | --- | --- | --- |
| 2. Degree, etc.  Subject    Natural Sciences  Medicine  Medicine | Degree BA  MB BChir  MD | | Class 1 | University  Cambridge  Cambridge  Cambridge | | Date  1962  1964  1968 |
| 3. Posts held (with dates: please identify tenure and source of funding of present post.  1980 - 1995 Director, MRC Laboratories, The Gambia  1995 - Professor of Communicable Disease and subsequently Manson Professor of Clinical Tropical Medicine, LSHTM | | | | | | |
| 4. Recent publications; also papers in press.   1. **Greenwood B M**, Alonso P (2002) Malaria vaccine trials. Chemical Immunology, 80, 366-395 2. **Greenwood B M**, Mutabingwa T (2002) Malaria in 2002. Nature, 415, 670-672 3. Miller L H, **Greenwood B M** (2002) Malaria – a shadow over Africa. Science, 298, 121-122 4. Chandramohan D, Jaffar S, **Greenwood B M** (2002) Use of clinical algorithms for diagnosing malaria. Tropical Medicine & International Health, 7 45-52. 5. **Greenwood B M** The molecular epidemiology of malaria. Tropical Medicine and International Health 7, 1012-1021 6. von Seidlein L, Clarke S, Alexander N, Manneh F, Doherty T, Pinder M, Walraven G, **Greenwood BM** (2002) Treatment uptake by individuals infected with *Plasmodium falciparum* in rural Gambia, West Africa. Bulletin of the World Health Organization, 80, 720-796. 7. Aucan C, Walley A J, **Greenwood B M**, Hill AVS, (2002) Haptoglobin genotypes are not associated with resistance to severe malaria in The Gambia. Transactions of the Royal Society of Tropical Medicine and Hygiene, 96, 327-328. 8. Sutherland C, Alloueche A, Curtis J, Drakeley C, Ord R, Durasingh M, **Greenwood B M**, Pinder M, Warhurst D, Targett G A T (2002) Gambian children successfully treated with chloroquine can harbour and transmit *Plasmodium falciparum* gametocytes carrying resistance genes. American Journal of Tropical Medicine and Hygiene, 67, 578-585 9. Alloueche A, Milligan P, Conway D J, Pinder M, Bojang K, Doherty T, Tornieport N, Cohen J, **Greenwood BM** (2003) Protective efficacy of RTS,S/ASO2 *Plasmodium falciparum* malaria vaccine is not strain specific. American Journal of Tropical Medicine and Hygiene 68, 97-101 | | | | | | |

| 1. Surname  Gyapong | | Forename(s)  John Owusu | | Age  d.o.b. 03/03/1962 | |
| --- | --- | --- | --- | --- | --- |
| 2. Degree, etc.  Subject  Human Biology  Medicine  Public Health in Developing countries  Public Health Epidemiology | Degree  B.Sc.  MB ChB  MSc  PhD | Class | University  School of Medical Sciences, University of Science and Technology, Kumasi, Ghana.  School of Medical Sciences, University of Science and Technology, Kumasi, Ghana.  London School of Hygiene and Tropical Medicine, University of London, England.  London School of Hygiene and Tropical Medicine, University of London, England. | | Date  1984  1987  1993  1997 |
| 3. Posts held (with dates: please identify tenure and source of funding of present post.  **Medical Officer**, Ministry of Health, Ghana, 1987-90: General duty medical officer responsible for outpatient and inpatient care in medicine, surgery, paediatrics, and obstetrics and gynaecology.  **Project Clinician**, Ghana Vitamin A Supplementation Trials 1990-92: Responsible for the clinical monitoring of children in a study to evaluate the impact of vitamin A supplementation on childhood morbidity and mortality in northern Ghana.  **Epidemiologist**, Navrongo Health Research Centre, Ghana, 1992-1994: Participate in the design and implementation of various studies in the research centre. Some of the studies were the social and economic impact of lymphatic filariasis, and the evaluation of the impact of permethrin-impregnated bednets on childhood mortality.  **Epidemiologist**, National Health Research Unit, Ghana. 1994-1997: Primarily responsible for training in health systems research in Ghana and conducting of various epidemiological studies. Played a key role in conducting in filariasis research that helped to shape the agenda for filariasis control at both the national and global level.  **Deputy Director,** National health Research Unit, 1997-2001  **Director**, National Health Research Unit, 2001-present | | | | | |
| 4. Recent publications; also papers in press.   1. Yeboah-Antwi K, **Gyapong JO**, Asare IK, Barnish G, Evans DB, and Adjei S (2001). The impact of pre-packaging of anti-malarial drugs on cost to patients and compliance to treatment. *Bulletin of the World Health Organisation,* **79:**394-399. 2. Ansah E, **Gyapong JO**, Agyepong IA, Evans DB (2001). Improving Adherence to malaria treatment for children: the use of pre-packed chloroquine tablets versus chloroquine syrup. *Journal of Tropical Medicine and International Health* 6(7): 496-504 3. **Gyapong JO** Remme JFH (2001). Validation of a rapid assessment method for the distribution of Bancroftian filariasis. *Transactions of the Royal Society of Tropical Medicine and Hygiene* **95:** 681-686. 4. Gyapong M, **Gyapong JO** and Owusu-Banahene G (2001). Community-directed treatment: the way forward to eliminating lymphatic filariasis as a public-health problem in Ghana. *Annals of Tropical Medicine and Parasitology*, **95**:77-86. 5. **Gyapong JO** (2000). Lymphatic filariasis in Ghana: from research to control. *Transactions of the Royal Society of Tropical Medicine and Hygiene* **94:** 599-601. 6. **Gyapong JO**, Kyelem D, Kleinschmidt I, Agbo K, Ahouandogbo F, Gaba J, Owusu-Banahene G, Sanou S, Sodahlon YK, Biswas G, Kale OO, Molyneux DH, Roungou JB, Thomson MC, Remme J. The use of spatial analysis in mapping the distribution of bancroftian filariasis in four West African countries. *Annals of Tropical Medicine and Parasitology* 2002 Oct;**96**(7):695-705. | | | | | |

| 1. Surname  CHANDRAMOHAN | Forename(s)  DANIEL | | Age 51 years old  d.o.b. 2 February 1952 | |
| --- | --- | --- | --- | --- |
| 2. Degree, etc.  Subject  MEDICINE  COMMUNITY HEALTH IN DEVELOPING COUNTRIES | Degree  MBBS  MSc | University  Madurai University, Madras, India  London University | | Date  1974  1992 |
| 3. Posts held (with dates: please identify tenure and source of funding of present post.   - 1. Medical Officer, Sacred Heart Hospital, Tuticorin, India   1976-1982 Medical Officer, Ministry of Health, Ethiopia  1983-1985 Medical superintendent, Mt Selinda Hospital, Zimbabwe  1986-1990 District Medical Officer, Chipinge district, Zimbabwe  1992-1995 Research Fellow, THEU, LSHTM  1996 to present Lecturer, DFID Malaria Programme, LSHTM | | | | |
| 4. Recent publications; also papers in press.   1. Owusu-Agyei S, Fryauff DJ, **Chandramohan D**, Koram KA, Binka FN, Nkrumah FK, Utz GC, Hoffman SL. Characteristics of severe anemia and its association with malaria in young children of the Kassena-Nankana District of northern Ghana. Am J Trop Med Hyg. 2002 Oct;67(4):371-7. 2. Rowland M, Webster J, Saleh P, **Chandramohan D**, Freeman T, Pearcy B, Durrani N, Rab A, Mohammed N. Prevention of malaria in Afghanistan through social marketing of insecticide-treated Nets: evaluation of coverage and effectiveness by cross-sectional surveys and passive surveillance. Trop Med Int Health. 2002 Oct;7(10): 813-22. 3. Hammerich A, Campbell OM, **Chandramohan D**. Unstable malaria transmission and maternal mortality-experiences from Rwanda. Trop Med Int Health. 2002 Jul;7(7):573-6. 4. **Chandramohan D**, Jaffar S, Greenwood B. Use of clinical algorithms for diagnosing malaria. Trop Med Int Health. 2002 Jan;7(1):45-52. 5. **Chandramohan D**, Carneiro I, Kavishwar A, Brugha R, Desai V, Greenwood B. A clinical algorithm for the diagnosis of malaria: results of an evaluation in an area of low endemicity. Trop Med Int Health. 2001 Jul;6(7):505-10. 6. **Chandramohan D**, Greenwood B, Cox J, Mswia R, Setel P. Relationship between malaria endemicity and acute febrile illness mortality in children. Bull World Health Organ. 2001;79(4):375-6.      1. Quigley MA, **Chandramohan D**, Rodrigues LC. Diagnostic accuracy of physician review, expert algorithms and data-derived algorithms in adult verbal autopsies. Int J Epidemiol. 1999 Dec; 28(6): 1081-7. 2. Brugha R, **Chandramohan D**, Zwi A. Viewpoint: management of malaria - working with the private sector. Trop Med Int Health. 1999 May;4(5):402-6. 3. **Chandramohan D**, Greenwood BM. Is there an interaction between human immunodeficiency virus and Plasmodium falciparum? Int J Epidemiol. 1998 Apr;27(2):296-301. | | | | |

| **Name** | **Code** |
| --- | --- |
| Seth Owusu-Agyei | OA |
| Brian Greenwood | BG |
| John Gyapong | JG |
| Daniel Chandramohan | DC |
| Kwaku Poku Asante | KP |
| Kojo Koram | KK |
| Mike Wilson | MW |
| Martin Adjuik | MA |
| Fred Binka | FB |
